# *In Vivo* Quantification Reveals Extensive Natural Variation in Mitochondrial Form and Function in *Caenorhabditis briggsae*

**DOI:** 10.1371/journal.pone.0043837

**Published:** 2012-08-28

**Authors:** Kiley A. Hicks, Dana K. Howe, Aubrey Leung, Dee R. Denver, Suzanne Estes

**Affiliations:** 1 Biology Department, Portland State University, Portland, Oregon, United States of America; 2 Department of Zoology and Center for Genome Research and Biocomputing, Oregon State University, Corvallis, Oregon, United States of America; University of Arkansas, United States of America

## Abstract

We have analyzed natural variation in mitochondrial form and function among a set of *Caenorhabditis briggsae* isolates known to harbor mitochondrial DNA structural variation in the form of a heteroplasmic *nad5* gene deletion (*nad5Δ*) that correlates negatively with organismal fitness. We performed *in vivo* quantification of 24 mitochondrial phenotypes including reactive oxygen species level, membrane potential, and aspects of organelle morphology, and observed significant among-isolate variation in 18 traits. Although several mitochondrial phenotypes were non-linearly associated with *nad5Δ* levels, most of the among-isolate phenotypic variation could be accounted for by phylogeographic clade membership. In particular, isolate-specific mitochondrial membrane potential was an excellent predictor of clade membership. We interpret this result in light of recent evidence for local adaptation to temperature in *C. briggsae*. Analysis of mitochondrial-nuclear hybrid strains provided support for both mtDNA and nuclear genetic variation as drivers of natural mitochondrial phenotype variation. This study demonstrates that multicellular eukaryotic species are capable of extensive natural variation in organellar phenotypes and highlights the potential of integrating evolutionary and cell biology perspectives.

## Introduction

Mitochondria are organelles that harbor DNA and produce most of the energy required to sustain eukaryotic life via an electron transport chain (ETC). Proper assembly and operation of the mitochondrial ETC relies upon the coordinated functioning of both nuclear and mitochondria-encoded subunits of ETC complexes. Hence, mutations affecting ETC genes can have a variety of detrimental consequences that manifest at cellular, tissue, and whole organism levels [1], [2], and have been implicated in many complex human diseases [3]–[6]. Proximal effects of ETC mutations include altering mitochondrial reactive oxygen species (ROS) production [7], [8], membrane potential (ΔΨM) [8]–[11], and other aspects of mitochondrial physiology. ROS are generated by the ETC as a byproduct of oxidative phosphorylation and are of particular interest because, when present at high levels, they can damage cellular macromolecules including mitochondrial and nuclear DNA and the components of the ETC itself [12]. Because ETC genetic variation can ultimately generate variation in organismal fitness through its effects on physiology, it is likely to be a significant target of natural selection. Accordingly, selection on ETC-dependent mitochondrial traits has been implicated in the evolution and diversification of life-history traits [13], [14], thermal tolerance [15]–[17], aging [18], reinforcement and allopatric speciation [19], [20], [21]–[24].

Despite the broad relevance of mitochondria to evolutionary processes and human health, understanding mitochondrial genotype-phenotype relationships has proven difficult [25]. One reason for this is the variable phenotypic expression of mitochondrial ETC mutations caused by variation in mitochondrial DNA (mtDNA) mutation heteroplasmy [26], [27]. Heteroplasmy occurs when both wildtype and mutant mtDNA genomes coexist within a mitochondrion, cell, tissue, or individual, and is a common characteristic of mtDNA mutations [28]. Heteroplasmy is the root cause of mitochondrial threshold effects wherein phenotypic consequences of mtDNA mutations only arise when heteroplasmy level reaches some threshold [26], [27]. For such reasons, we still have a limited understanding of the causes and consequences of ETC mutations *in vivo*, and virtually no such information on naturally occurring heteroplasmic ETC mutations. To date, most studies have been conducted on isolated mitochondrial fractions, chemically treated cell lines or yeast strains, or on those containing experimentally generated mutations [29].

Advances in biological imaging techniques have facilitated recent *in vivo* studies of mitochondria and are permitting unprecedented views into the dynamic structure of mitochondria and mitochondrial populations. Regular cycles of fission, fusion, and degradation are now known to maintain organellar populations and contribute to spatiotemporal variation in mitochondrial morphology [30], [31]. Recent studies have identified and characterized genes that control mitochondrial fission and fusion [32]–[36]. In addition to these loci, mutations in a number of ETC genes also result in abnormal mitochondrial morphology [37]–[39]. It is increasingly apparent that mitochondrial shape and function are intimately linked such that changes in morphology can affect diverse processes such as free radical signaling, energy metabolism [32], [39]–[43], and cell cycle regulation [44]. However, the physiological causes of altered mitochondrial shape and the effects of organellar morphology on organellar function are poorly known. Further, we have no information regarding standing levels of variation in these mitochondrial traits or its evolutionary consequences.

**Figure 1:**
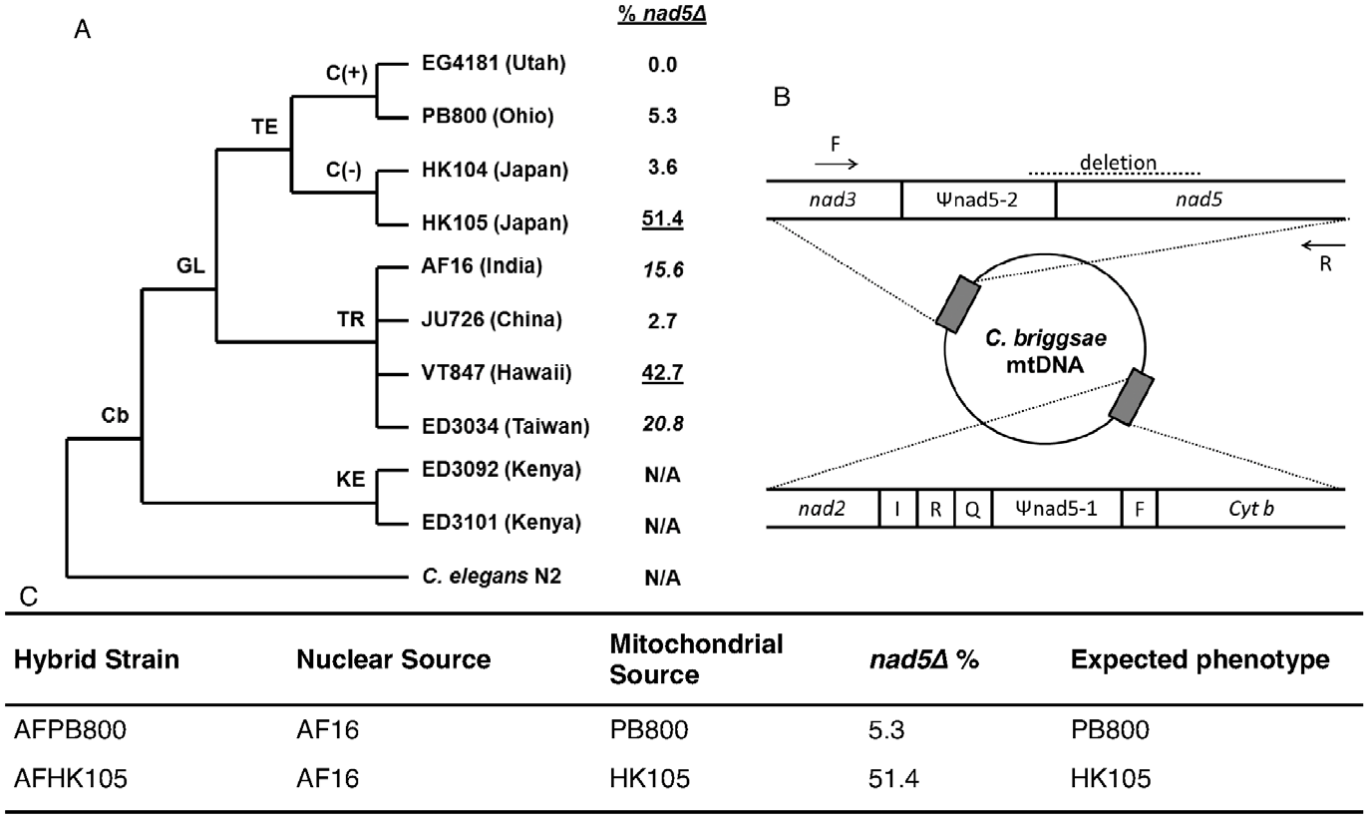
Natural and experimental *C. briggsae* strains and description of the *nad5Δ* mtDNA deletion. A. Phylogenetic relationship and *nad5Δ* heteroplasmy level of *C. briggsae* isolates studied here. GL = global superclade; KE = Kenya clade; TE and TR = temperate and tropical subclades of GL; C(+) = isolates bearing compensatory Ψ*nad5Δ*-2 allele; C(-) = isolates bearing ancestral alleles. *nad5Δ* heteroplasmy categories were assigned to each *C. briggsae* natural isolate for statistical analysis following Estes et al. (2011): High = underlined font, medium = italicized, low = regular, and zero-*nad5Δ*="N/A”. Note that we assayed the natural HK104 isolate here instead of the mutation-accumulation line progenitor reported in Estes et al. (2011), which had evolved high *nad5Δ* levels in the lab (See Materials and Methods). B. Positions of the *nad5Δ* deletion (dashed line at top) and Ψ*nad5Δ*-2 elements in the mitochondrial genome. Primers are indicated by arrows (adapted from Howe and Denver, 2008). C. Mitochondrial and nuclear parent isolates for each mitochondrial-nuclear hybrid. *nad5Δ* heteroplasmy for each hybrid strain matches that of the maternal isolates as expected. Mitochondrial phenotypes are expected to match those of the maternal isolate if measured traits are predominantly determined by the mitochondrial genotype.

Recently, geographically diverse isolates of *C. briggsae* nematodes have been studied with the aim of developing a model for natural population genetic and genomic studies [45]–[48]. Like *C. elegans*, *C. briggsae* is globally distributed and offers many of the same advantages as an experimental system [49] however, *C. briggsae* exhibits higher rates of mutation [50], [51], greater molecular genetic diversity, and more extreme population subdivision than *C. elegans*. Nuclear [46] and mitochondrial [52] genetic analyses have identified four major phylogeographic clades of *C. briggsae* (the distal three of which are shown in Fig. 1), which may be adapted to local thermal regimes [53], [54] and other conditions. Howe and Denver (2008) discovered that many isolates of this species contain varying levels of a large, heteroplasmic mtDNA deletion named *nad5Δ* (Fig. 1A and further described in Materials and Methods). The deletion removes nearly half of the *NADH-dehydrogenase 5* (*nad5*) gene (Fig. 1B), which encodes a central subunit of mitochondrial ETC complex I. *nad5Δ* appears to occur via illegitimate recombination events between the *nad5* gene and an upstream pseudogene, Ψnad5–2, derived from *nad5*. The “Kenya” (aka Nairobi) *C. briggsae* clade does not contain the pseudogene and therefore does not experience the deletion, while other *C. briggsae* isolates exhibit *nad5Δ* heteroplasmy levels ranging from zero to over 50%. A number of findings suggest that *nad5Δ* might be detrimental for several aspects of nematode health and fitness – especially when heteroplasmy levels exceed ∼40% [47], [52] - as expected based on studies of *nad5* and other complex I mutants [55]–[60]. Together with recent work showing that *nad5Δ* behaves as a selfish genetic element; i.e., experiences a strong transmission bias [48], these results indicate that isolate-specific *nad5Δ* levels are probably shaped by a combination of evolutionary forces: recurrent mutation and deletion transmission biases, genetic drift due to sampling of mitochondria during fertilization, and truncation selection.

Here we expanded our previous work to analyze variation in *C. briggsae* mitochondrial form and function using natural and experimental hybrid populations of nematodes. This study provides an assessment of natural levels of subcellular phenotypic variation and takes a first step toward understanding how this species’ extensive ETC genetic variation may affect cellular traits *in vivo*. We identified traits that diagnose mitochondrial dysfunction and hypothesized that *C. briggsae* isolates with higher *nad5Δ* levels would exhibit mitochondrial phenotypes often associated with such dysfunction, including increased ROS, decreased ΔΨM, and more fragmented mitochondria. However, we found that several mitochondrial phenotypes were non-linearly associated with *nad5Δ* level and that much of the among-isolate phenotypic variation was best explained by phylogeographic clade membership rather than *nad5Δ* frequency. Analysis of mitochondrial-nuclear hybrid strains provided support for both mtDNA and nuclear genetic variation as drivers of natural mitochondrial phenotype variation.

## Materials and Methods

### Nematode Strains and Culture Conditions

For the current study, we used ten natural *C. briggsae* isolates and two experimental hybrid strains derived from three of these isolates (Fig. 1). The ten isolates represent three of the four major phylogeographic clades of *C. briggsae*, encompass the known range of *nad5Δ* heteroplasmy level, and were chosen to include two isolates that do not experience the deletion and two isolates containing a putative compensatory mutation that limits *nad5Δ* formation [47], [52]. We note here that we assayed a different strain of HK104 from that analyzed in Estes et al. (2011). Here we study the natural HK104 isolate, which has low *nad5Δ* (Fig. 1), while Estes et al. (2011) used an inbred HK104 line that had evolved higher *nad5Δ* frequency. Briefly, *nad5Δ* segregates among natural populations of *C. briggsae* and requires the presence of the Ψnad5-2 pseudogene (Fig. 1B). Sequence repeats within *nad5Δ* and Ψnad5-2 promote direct repeat-associated deletion events. Isolates in the Kenya clade (Fig. 1A) lack the pseudogene and are therefore spared *nad5Δ*. Among-isolate variation in *nad5Δ* heteroplasmy level is also controlled by the presence of compensatory sequences within the mtDNA of certain isolates [47], which appear to place an upper bound on the proportion of *nad5Δ-*deletion bearing genomes able to accrue within individuals [52]. *nad5Δ* was recently shown to behave as a selfish genetic element that increases in frequency when *C. briggsae* isolates are maintained by single-individual bottlenecking [48]; however, *nad5Δ* heteroplasmy level is stably maintained across generations when isolates are maintained in larger population sizes (N∼100) where natural selection is more efficient (Estes, Coleman-Hulbert, Howe, and Denver, unpubl. data). Great care was taken to ensure that *C. briggsae* natural isolates did not experience population bottlenecks in the lab and *nad5Δ* heteroplasmy levels were found to remain stable over the course of our study [47].

To test the relative contributions of mitochondrial and nuclear genomes to among-isolate phenotypic variability, we also studied two mitonuclear hybrid strains that contain the mitochondrial genome of one isolate (PB800 or HK105) upon the same (AF16) nuclear genetic background (Fig. 1C). These strains were generated through serial backcrossing of AF16 males to hermaphrodite cross-progeny for 10 generations. This process is expected to result in worms with >99.9% of their nuclear genome from AF16 and their mitochondrial genome from a separate *C. briggsae* isolate. We confirmed that each hybrid line encoded the expected mtDNA through PCR and direct sequencing of a portion of *nad5Δ* [52] and *COII* [61] genes. Each hybrid line was also confirmed to harbor *nad5Δ* levels similar to that of the original hermaphrodite mitochondrial donor parent through agarose gel analysis of PCR reactions using primers that flank the deletion region as described in Howe and Denver (2008) (Fig. 1C).

Nuclear contributions of hybrid lines were confirmed by evaluating six nuclear PCR loci, one for each of the six *C. briggsae* chromosomes, that contained single nucleotide polymorphism or large-indel variants between the two strains involved in the crosses. SNP variants were evaluated by fluorescent capillary DNA sequencing and large-indel variants were evaluated by agarose gel electrophoresis. The loci used were taken from published variants described in [62], [63]. For the AF16 x HK105 hybrid lines (expected to contain AF16 nuclear DNA), 6/6 target loci contained differences between the strains and 6/6 of the loci were confirmed to contain only the AF16 sequence in the hybrid line. For the AF16 x PB800 cross (also expected to contain AF16 nuclear DNA), only 5/6 loci had diagnostic sequence differences between the two strains; the chromosome V locus employed was identical between AF16 and PB800. However, 5/5 of the diagnostic nuclear loci confirmed the presence of the AF16 sequence only in the hybrid strain.

We note here that we attempted to generate additional sets of mitonuclear hybrid strains by crossing other pairs of *C. briggsae* natural isolates; however, molecular genetic assays revealed that bi-parental inheritance of mitochondrial genotypes had occurred in these lines. In a similar study involving inter-strain crosses in *C. elegans*, mitochondrial DNA was observed to be strictly maternally inherited (W.K. Thomas and K. Morris, unpubl. data). Thus, our result may suggest that favorable mitonuclear epistatic interactions specific to individual *C. briggsae* isolates were disrupted in some hybrid strains such that paternal mitochondria were transmitted to hybrid offspring [64]–[67]. Such a situation might arise if hybridization in some way disrupts the autophagic degradation of paternal mitochondria that normally occurs after fertilization [68], [69], for example. These strains were therefore omitted from the current analysis.

Nematodes were grown under standard laboratory conditions at 25°C on 15 mm NGM Petri plates seeded with HB101 *Escherichia coli* as a food source. All strains were included in each analysis. Prior to each assay, age synchronous worms were obtained through a standard bleaching protocol.

### Fluorescence Microscopy

**Figure 2:**
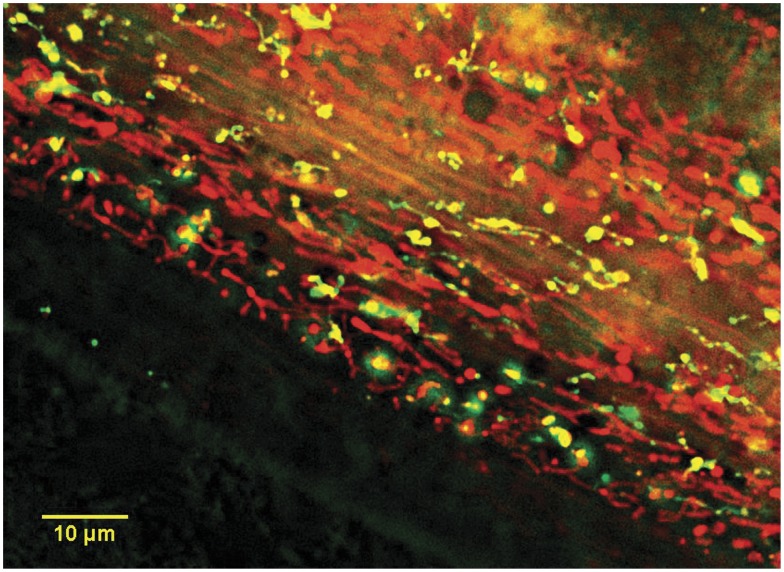
Localization of mitochondria-targeted dyes in *C. briggsae* nematodes. A Z-projection image of MitoTracker Red CMXRos (red objects) and MitoTracker Green FM (green objects) specific staining, and colocalization of these probes (yellow objects) within an individual (after processing to find edges of objects). Green objects are relatively depolarized mitochondria that have taken up primarily MitoTracker Green FM probe; red objects are more polarized mitochondria that have taken up mostly MitoTracker Red probe. Yellow objects likely have intermediate polarization and have taken up equivalent amounts of each probe. Scale bar = 10 µm.

We performed confocal image analysis on young adult nematodes treated with mitochondria-targeted fluorescent dyes. Fluorescent imaging provides the distinct advantage of allowing simultaneous localization and relative quantification of mitochondrial traits (Fig. 2). Additionally, recent work indicates that ROS data obtained from fluorescent analysis and electron spin resonance yield similar results [70]. This dye-based method has received criticism for its potential to be influenced by variable feeding rates [71]. Importantly, we find no correlation between pharyngeal pumping rates [47] and maximal ROS or ΔΨM as measured here (ROS ρ = −0.024, p = 0.955; ΔΨM ρ = 0.167, p = 0.693). Also, unlike dye-based methods performed using whole-worm lysate (B. Halliwell, pers. comm.), our method does not cause the disruption and release of organellar and intracellular contents, which can lead to increased ROS via the release of free iron and ensuing Fenton reactions [72]. Prior to each assay, age synchronous worms were incubated with *E. coli* labeled with 10 µM concentrations of the specific fluorescent dye(s) appropriate for each experiment. Concurrently, a second age synchronous batch of worms from the same strain was incubated without dye to serve as the control. After 24 hours of exposure, young adult stage worms were washed and transferred to fresh NGM plates containing unlabeled *E.coli*. Worms were allowed to feed for one hour, which clears the digestive tract of any labeled *E. coli* that could interfere with accurate fluorescence measurements. Immediately prior to imaging, worms were paralyzed following the methods of Dingley et al. (2009) with a single drop of 5 M levamisole (Molecular Probes Inc., Eugene, OR, Carlsbad, CA), which immobilizes worms by preventing depolarization of skeletal muscle [73].

For each dye-based assay, images of the pharynx of each nematode were collected. The pharynx is a neuromuscular organ that nematodes use to ingest bacteria [74]. We chose to analyze pharyngeal bulbs for two reasons: 1) they are easily visualized and have high levels of dye uptake, which serves to reduce technical variation, and 2) they are mitochondria-rich but contain few lipid droplets, which are highly autofluorescent and thus interfere with accurate measurement of mitochondrial fluorescence. Images were acquired using a high resolution wide field Core DV system (Applied Precision™, Issaquah, WA) equipped with an Olympus IX71 inverted microscope mounted with a Nikon Coolsnap ES2 HQ camera (Advanced Light Microscopy Core Facility, Oregon Health and Science University, Portland, OR). Fluorescent z-stack images of the pharyngeal bulb of individual worms were captured at 60X magnification using a short arc 250W Xenon lamp. All images were captured immediately following paralysis. Images were optimized by deconvolving, and relative fluorescence values were obtained using ImageJ software (NIH, Bethesda, MD).

### Relative Mitochondrial Oxidant Levels

ROS levels were assessed *in vivo* for all *C. briggsae* isolates following the basic approach of [8] and described further in Estes et al. (2011). Briefly, worms were incubated for 24 hours in 10 uM MitoSOX Red (Molecular Probes Inc., Eugene, OR) before imaging as outlined above. MitoSOX Red was recently shown to quantify total levels of mitochondrial oxidants, rather than superoxide specifically, when used with confocal microscopy [75]. Images of the pharynx were captured with a 1.0 second exposure time. Terminal pharyngeal bulbs were manually encircled within each image to quantify mean fluorescence intensity of the area in exposed and unexposed (control) animals from each strain. The final pharyngeal bulb intensity values were calculated as the difference between intensity values for exposed and control worms. We note here that we have previously measured ROS levels for most of the natural isolates studied here [47] and that the results presented here are the combined data from the current study and the previous study. In the current study, however, we have doubled our sample size (20 individuals) and have included additional strains in the analysis: two mitonuclear hybrid strains, one isolate with an intermediate *nad5Δ* frequency level, ED3034, and the natural isolate of HK104 (Fig. 1A). All other isolates performed the same in each assay with respect to ROS level (t ≤1.77, p≥0.098); we therefore combined the ROS data from the two assays to increase statistical power.

The efficacy of a second mitochondrial ROS detecting dye, MitoTracker Red CM-H_2_XRos (Molecular Probes Inc., Eugene, OR), was assessed using the same protocol as above. We initially believed that this dye would be preferable over MitoSOX Red because of its greater sensitivity to ROS [70]. However, although the fluorescent signal from MitoTracker Red CM-H_2_XRos-treated mitochondria was distinct, the background fluorescence generated from non-specific lipid uptake of the dye decreased the precision of the fluorescence measurement [76]. MitoSOX Red generated less background fluorescence and produced more precise images, and was therefore used for mitochondrial ROS detection.

### Relative Mitochondrial ΔΨM

Relative ΔΨM was assessed using MitoTracker Red CMXRos, the uptake of which is dependent upon mitochondrial ΔΨM. We utilized the ROS assessment protocol (above), except that a shorter, 0.08 seconds, exposure time was used for imaging; these assays were performed concurrently with those for mitochondrial morphology (below).

### Mitochondrial Morphology

Quantification of differences in mitochondrial morphology was achieved by co-labeling worms with MitoTracker Green FM, which is not ΔΨM-specific, and MitoTracker Red CMXRos, which accumulates exclusively within actively respiring mitochondria (Fig. 2). This allowed us to distinguish between polarized and depolarized mitochondria. Worms were prepared for imaging as above, this time with exposures of 0.08 seconds for MitoTracker Red and 0.02 seconds for MitoTracker Green.

We also confirmed that altered ΔΨM would produce the expected effects on mitochondrial morphology [32] by depleting ΔΨM with 10 µM carbonylcyanide-3-chlorophenylhydrazone (CCCP, Sigma-Aldrich, St. Louis, MO) in 15 individuals from mid-deletion isolate, ED3034 (Fig. 1A). Beginning at the L1 stage, worms were transferred daily to fresh CCCP-treated plates until they reached young adulthood. Compared to an equal number of untreated ED3034 individuals, we found that CCCP treatment reduced the size of both the total and the functional (polarized) mitochondrial population (A_TP_: F = 6.185, p = 0.0202, A_FP_: F = 7.607, p = 0.0134), the size of individual functional mitochondria (A_F_: F = 4.591, p = 0.0425), and the variance in circularity of functional mitochondria (C_FV_: F = 7.136, p = 0.0134) – as expected if organelles are becoming increasingly fragmented. (See below and Table 1 for further explanation.).

**Table 1:** Assigned labels and descriptions of all mitochondrial traits measured for *C. briggsae* natural isolates.

Label	Trait	Description	Grand mean	F
				df
*Measures of Mitochondrial Physiology*				
ΔΨM Mean	Membrane potential	Average of mean relative MitoTracker Red CMXRosfluorescence	836.8	90.99***
				182, 9
**ΔΨM Max**		**Average of max relative MitoTracker Red CMXRos fluorescence**	**2331**	75.20***
				**182, 9**
ROS Mean	Reactive Oxygen Species	Average of mean relative MitoSOX Red fluorescence	298.0	9.269***
				201,9
**ROS Max**		**Average of max relative MitoSOX Red fluorescence**	**1643**	12.18***
				**201, 9**
*Measures of the Mitochondrial Population*				
**A_FP_**	Area of mitochondrial population	Area of functional, non-functional or total (both functionaland non-functional) mitochondrial populations	**2870**	4.923***
				**169, 9**
**A_NP_**			**3152**	2.012*
				**169, 9**
A_TP_			6022	3.349***
				169, 9
A_FP/NP_	Ratio of functional to non-functional mitochondrial area	Area of the functional mitochondrial population/area of thenon-functional population	1.151	3.703***
				169, 9
A_FP/TP_	% functional area	Area of the functional mitochondrial population/area of thetotal population	0.443	3.434***
				169, 9
N_F_	Number of mitochondria	Number of functional, non-functional, or total individual mitochondria	67.91	6.247***
				169, 9
N_N_			58.85	2.143*
				169, 9
N_T_			124.7	3.977***
				169, 9
**N_F/N_**	**Ratio of functional to non-functional mitochondria**	**Number of functional mitochondria/number of** **non-functional mitochondria**	**1.331**	3.451***
				**169, 9**
N_F/T_	% functional mitochondria	Number of functional mitochondria/number of total mitochondria	0.505	3.983***
				169, 9
*Measures of Individual Mitochondrial Shape*				
A_F_	Area of individual mitochondria	Average area of individual functional or non-functional mitochondria	40.74	1.504
				169, 9
**A_N_**			**55.70**	2.284*
				**169, 9**
AR_F_	Aspect ratio	Average of the ratio between the major and minor axisof the ellipse equivalent to each functional or non-functional mitochondrion	1.669	1.499
				169, 9
**AR_N_**			**1.865**	5.232***
				**169, 9**
AR_FV_	Aspect ratio variance	Average within-individual variance in aspect ratio offunctional or non-functional mitochondria	0.655	1.875
				163, 9
AR_NV_			1.271	1.348
				166, 9
C_F_	Circularity	4π (area/perimeter^2^) for functional or non-functional mitochondria	0.8619	1.771
				169, 9
**C_N_**			**0.825**	3.494***
				**169, 9**
C_FV_	Circularity variance	Within-individual variance in circularity of functional or non-functional mitochondria	0.037	1.891
				163,9
**C_NV_**			**0.052**	2.049*
				**166, 9**

The grand mean, F-ratio and degrees of freedom for one-way ANOVA testing for phenotypic differences among *C. briggsae* isolates. Bold font identifies the nine traits retained in the classification tree analysis when using categories based on isolate-specific *nad5Δ* % (see Table S3). *, **, and *** denote p<0.05, 0.01, 0.001, respectively. Subscripts N, F, and T indicate whether the measure refers to Non-functional, Functional, or Total mitochondria. Subscript P and V denote that the measure refers to the entire mitochondrial population (not individual mitochondria), or the average individual variance in that trait, respectively.

### Mitochondrial Localization of Fluorescent Dyes and Effect of Levamisole

We observed extensive localization of MitoTracker Green FM and Red CMXRos (Fig. 2), which points to mitochondria-specific staining by fluorescent dyes. This specificity was further confirmed by depleting ΔΨM using CCCP (as above) and directly visualizing reductions in the fluorescence intensity of all probes used, especially the membrane-potential dependent dyes (data not shown). We also tested the effect of levamisole, the drug used to paralyze worms prior to imaging, on dye fluorescence using five age synchronous individuals from isolates PB800 and HK105 (Fig. 1). There was no significant effect of levamisole on the mean or maximum fluorescence values of MitoTracker Red CMXRos or MitoTracker Green FM, nor was there a levamisole-by-isolate interaction. Similarly, levamisole had no effect on the maximum values of MitoSOX fluorescence (t-tests, p>0.291); however, there were significant effects of levamisole (t = −2.29, p = 0.038) and the interaction of levamisole and isolate (t = −2.13, p = 0.051) on mean MitoSOX fluorescence. Maximum fluorescence values were therefore used for all statistical analyses so that any isolate-by-probe interactions generated by levamisole were unlikely to influence our among-isolate comparisons. Furthermore, maximum measures are more consistent in fluorescence image analysis because they are unaffected by variation in pixel size and mitochondrial number or area between images.

We also attempted to co-label nematodes treated as above with either DAPI or Hoechst 33342 (Sigma) in order to visualize cell nuclei, which would have allowed us to assess the intracellular distribution of mitochondria. (Appropriate GFP fusions are not yet available for *C. briggsae*.) Unfortunately, both DAPI and Hoechst noticeably interfered with the fluorescence of the above MitoTracker dyes in *C. briggsae* (Hicks, pers. obs.). Our study therefore focuses on properties of individual mitochondria and mitochondrial populations within the pharyngeal tissue.

### Image Analysis

All image analyses were performed in ImageJ (NIH, ver. 1.43 u). Quantification of relative ROS and ΔΨM levels was achieved by manually enclosing the terminal pharyngeal bulb of each image to find the average intensity of the area as described in [8]. Mitochondrial morphology traits were quantified by processing images following [77]. Briefly, visibility of MitoTracker stained structures was improved by applying a linear stretch of the pixel intensity histogram corresponding to each slice in the z stack. This process enhances the contrast of an image by adjusting the number of low and high intensity pixels in the image based on the lowest and highest pixel values in the current image [78]. The image was then converted into a z-projection, a process that effectively removes the spaces between each slice of the z-stack creating a composite image from all slices. A 7×7 top-hat filter was then applied, followed by a median filter and a thresholding step [77]. The thresholded image was then converted to a binary image, which results in white mitochondria on a black background that can be analyzed in ImageJ.

To quantify among-isolate differences in mitochondrial form and function, we defined and measured 24 traits (Table 1). We evaluated functionality of mitochondria based on relative membrane potential (ΔΨM mean, ΔΨM max) and reactive oxygen species (ROS mean, ROS max). We also quantified various features of the mitochondrial population: the combined area of the mitochondrial population (A_FP_, A_NP_, A_TP_), the ratio of the area of functional to non-functional mitochondria (A_FP/NP_), and the percentage of the total mitochondrial area that is functional (A_FP/TP_), the number of organelles (N_F_, N_N_, N_T_), the ratio of functional to non-functional organelles (N_F/N_), and the percentage of functional mitochondria (N_F/T_), as defined by uptake of MitoTracker Red CMXRos. To describe organellar shape differences we quantified the area (A_F_, A_N_), aspect ratio (AR_F_, AR_N_), and circularity (C_F_, C_N_) of individual mitochondria. Aspect ratio measures the ratio between the long and short axes of an ellipse fit to the object in question [78]. It has a minimal value of 1, which corresponds to a perfect circle. Circularity (sometimes referred to as formfactor in the literature) is calculated as 4Π(area/perimeter^2^), and will also equal 1 when the measured object is a perfect circle. As the object becomes more elongated and/or branched, circularity approaches 0 [78]. Because circularity cannot accurately be measured for extremely small objects (ImageJ website), we omitted from all analyses mitochondria smaller than 2 pixels (or 0.129 microns). The number of such objects removed from analyses was small (ranging from 0–20 with most <10) and did not differ among isolates (F <1.618, p>0.094); this procedure likely had no impact on our phenotypic comparisons among isolates. Finally, to examine heterogeneity in mitochondrial form we estimated within-individual variance in aspect ratio (AR_FV_, AR_NV_) and circularity (C_FV_, C_NV_) of mitochondria for all strains.

### Statistical Analysis

Classification trees were used to determine which mitochondrial characteristics most accurately grouped *C. briggsae* isolates into categories corresponding to *nad5Δ* heteroplasmy level or to phylogeographic clades. A classification or decision tree is a data reduction technique that predicts the membership of data points within classes of a categorical “dependent” variable [79]. We chose the classification tree method over PCA or discriminant analysis because the latter techniques assume linearity and equal variance among groups, which our data violated. We first performed one-way analyses of variance (ANOVA) for each of the 24 mitochondrial traits to determine which of them varied significantly among the natural isolates. (Non-parametric analyses gave the same results and are not presented.) Eighteen of 24 traits exhibited significant among-isolate variation (Table 1) and were retained as descriptor variables in the classification tree analyses. For the first analysis, the ten natural isolates were grouped into four categories corresponding to their relative levels of *nad5Δ* frequency as before [47]. Classification trees were run five times for both analyses. For each run, traits that were retained in the tree were recorded along with the misclassification rate and R^2^ value. We considered any trait retained in 4 of 5 runs to be important under that scheme.

Analysis of among-isolate or hybrid strain variation was performed using separate one-way analyses of variance (ANOVA) for each phenotype measured (above). Least-squares contrasts (Tukey’s HSD for all pairwise comparisons) were used to test for differences between pairs of isolates. Additionally, we tested the effect of phylogenetic clade and assessed within-clade variation using nested ANOVA for each trait. To test for associations between traits and *nad5Δ* levels in *C. briggsae,* each trait was regressed onto isolate-specific *nad5Δ* percentages. All analyses were performed in JMP 9 (SAS Institute, Cary, NC).

## Results

### Natural Variation in Mitochondrial Phenotypes

**Figure 3:**
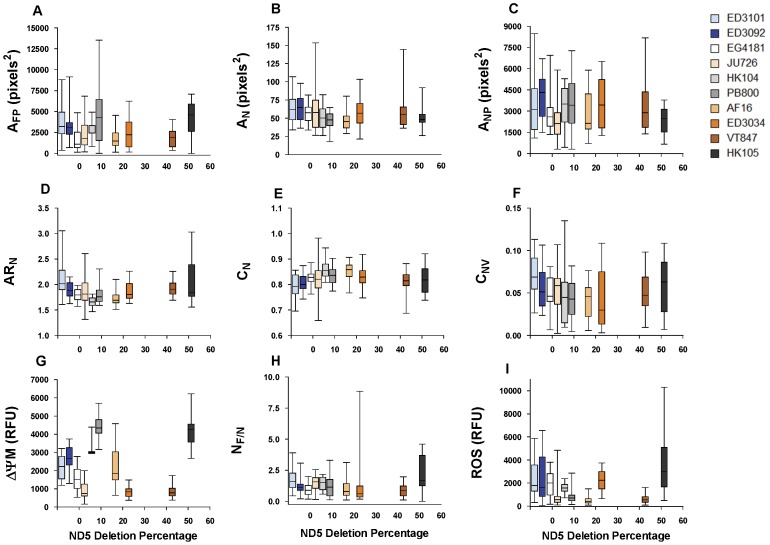
Associations between mitochondrial function and morphology traits and isolate-specific *nad5Δ* level. Natural variation among *C. briggsae* isolates in (A) the total area of functional mitochondria, (B) the average area of individual non-functional mitochondria, (C) the total area of non-functional mitochondria, the (D) aspect ratio, (E) circularity, (F) circularity variance of non-functional mitochondria, in (G) relative ΔΨM, (I) the ratio of functional to non-functional organelles, and (H) relative ROS levels. Column colors corresponding to phylogenetic clade (orange = Kenya, white = Temperate, blue = Tropical), and isolates are ordered by deletion frequency along the x-axis. ED3101 and ED3092 do not experience the deletion and were assigned arbitrary x-values of −7 and −5, respectively, for this figure. Averages of maximum pharyngeal bulb fluorescence in *C. briggsae* natural isolates are plotted in relative fluorescence units (RFU). Bars represent one SEM for 15–20 independent samples.

We quantified natural variation in 24 phenotypes that describe mitochondrial function and shape (Table 1) among ten distinct *C. briggsae* isolates (Fig. 1A). A majority (18 of 24) of the measured traits exhibited significant among-isolate variation. Mitochondrial ΔΨM showed the greatest among-isolate diversity, followed by mitochondrial ROS levels (Table 1; Fig. 3). Notably, mitochondria that we considered to be functional (i.e., polarized) by virtue of their having sufficient ΔΨM to permit uptake of MitoTracker Red CMXRos (Fig. 2; Materials and Methods) did not vary significantly among isolates in shape or heterogeneity in shape (Table 1). In contrast, characteristics of shape and heterogeneity in non-functional (depolarized) mitochondria often differed significantly among isolates. Traits that describe features of mitochondrial populations (i.e., all ten traits that describe the combined area or number of mitochondria), however, differed among isolates for both functional and non-functional mitochondria (Table 1).

We also found striking variation among phylogeographic clades of *C. briggsae* (Fig. 3; Table S1). Of the nine traits that best distinguished isolates with different *nad5Δ* levels (see below), all but N_F/N_ – the ratio of the number of functional to non-functional organelles – varied significantly among *C. briggsae* clades, consistent with a phylogenetic effect on many mitochondrial traits. In all of these cases, among-clade variation exceeded the variation observed within clades (Table S1). To further explore among-clade phenotypic differences, we used classification tree analysis to identify which of the 24 measured mitochondrial traits best distinguished three major phylogeographic clades of *C. briggsa*e (Table S2). Classification trees invariably retained only ΔΨM in the analysis. Misclassification rates were somewhat high (34%); isolate-specific ΔΨM nonetheless accounted for 20% of the total variation among clades. Further, if we allowed classification trees to continue splitting beyond the optimal number of splits (i.e., when further partitioning failed to account for a significant fraction of the total variation), analyses again used ΔΨM to further classify groups of isolates.

### Possible Relationships between *nad5Δ* and Phenotypes

We also used classification trees to determine whether mitochondrial traits distinguished groups of *C. briggsae* isolates with different *nad5Δ* heteroplasmy levels (Fig. 1A). Classification trees invariably used ROS and ΔΨM to classify *nad5Δ* frequency groups (Table S2). In addition to ROS and ΔΨM levels, the phenotypes most often used to classify *nad5Δ* categories were the area of non-functional and functional mitochondrial populations (A_NP_, A_FP_), descriptors of non-functional mitochondrial shape (A_N_, AR_N_, C_N_, C_NV_), and the ratio of functional to non-functional mitochondria (N_F/N_) (Table S2). About 45% of the total phenotypic variation was accounted for, but misclassification rates were again fairly high (33% on average), meaning that these traits were imperfect predictors of *nad5Δ* category.

As a second means of testing for any association between mitochondrial phenotypes and *nad5Δ* frequency in *C. briggsae,* we examined individual relationships between all measured traits and isolate-specific *nad5Δ* level. A second order quadratic provided the best fit of many traits to *nad5Δ* percentage (Fig. 3; Table S3); however, only a minimal amount of the total variation in these traits could be attributed to *nad5Δ* level (low R^2^ values in Table S3). For instance, zero- and high-*nad5Δ* isolates exhibited the highest values for mitochondrial ROS and ΔΨM; however, the relationship between these traits and *nad5Δ* is weak (Fig. 3; Table S3). All mitochondrial traits describing non-functional organelle shape (AR_N_, C_N_, C_NV_) exhibited stronger non-linear (quadratic) relationships with *nad5Δ* level, as did both measures describing the functional mitochondrial population (A_FP_, N_F/N_) (Fig. 3; Table S3). Neither the mean area of individual non-functional mitochondria (A_N_) nor the combined area of these mitochondria (A_NP_) was significantly associated with *nad5Δ* percentage in this analysis. However, non-functional mitochondria (A_N_) were larger on average than functional mitochondria (A_F_) (Table 1). Overall, *nad5Δ* load was more strongly associated with descriptors of the shape of non-functional mitochondria and traits describing the total population of functional mitochondria than with those describing any aspect of functional mitochondrial shape.

### Insights from Mitochondrial-nuclear Hybrid Lines

**Figure 4:**
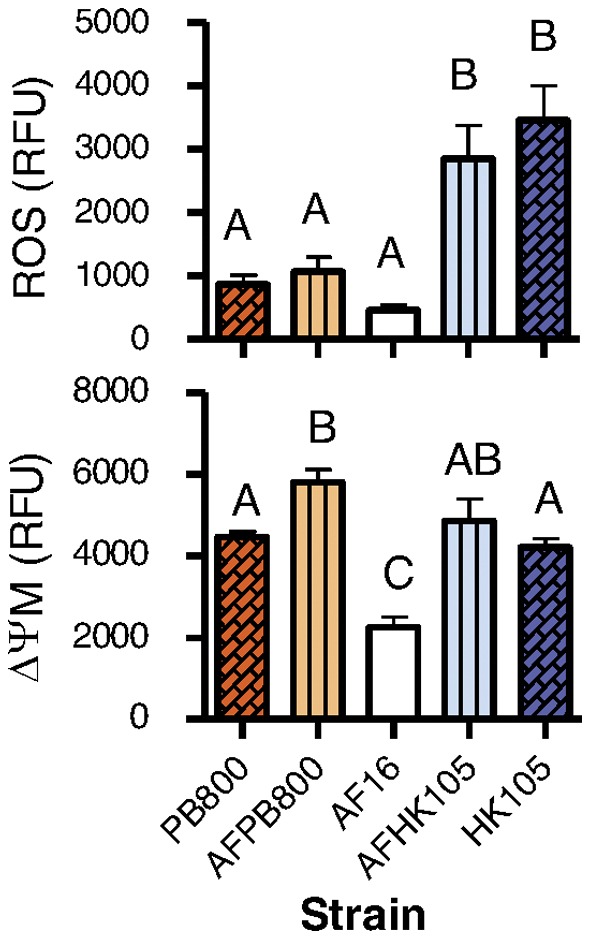
Mitonuclear hybrid strains more often resemble their mitochondrial parental isolate. Averages of maximum pharyngeal bulb fluorescence for mitochondrial (PB800 and HK105) and nuclear (AF16) parent isolates are on either side of the two hybrid strains (AFPB800 and AFHK105) (Fig. 1). Letters denote significantly different groups as determined by Tukey HSD analysis. Bars show one SEM for 15–20 independent samples.

To directly test whether the observed differences among *C. briggsae* isolates were due to mtDNA variation, we examined the nine phenotypes retained in classification tree analyses on two mitochondrial-nuclear hybrid strains (e.g., Fig. 4). If among-isolate variation in mitochondrial phenotypes is primarily due to an additive mtDNA genetic contribution, we predicted a correspondence between mitochondrial-nuclear hybrid strain phenotypes and their respective mitochondrial parental strain. Consistent with this idea, we observed that hybrid strains were more similar to their mitochondrial parent strains than to their nuclear parent strains (Table S4). Considering ROS and ΔΨM only, the hybrid strains were more similar to their mitochondrial parent in each case (Fig. 4). Note that in the AF16 and PB800 pairing, the hybrid is not significantly different from the nuclear parent, but is still more similar to the mitochondrial parent. The diversity in ROS and ΔΨM phenotypes seen in the three strains studied therefore appears to be due to variation in mtDNA as opposed to among-strain nuclear variation. However, the relative contribution of each genome was less clear for measures of the mitochondrial population and mitochondrial shape. These traits rarely differed significantly among parent and hybrid strains studied here (Table S4), although a larger study may have revealed small differences. A visual inspection of these data suggested that mitochondrial morphology traits may be more affected by the nuclear genome (hybrids are more similar to the paternal isolate) or by mitochondrial-nuclear epistasis (hybrids differ from both parental isolates), than were ROS and ΔΨM (not shown).

## Discussion

### Evolutionary Implications

We have used *in vivo* techniques with *C. briggsae* nematodes to conduct the first analysis of naturally-occurring variation in mitochondrial function, morphology, and properties of mitochondrial populations. We discovered considerable variation among natural *C. briggsae* isolates for most of the measured traits. In fact, we find more variation among natural isolates of *C. briggsae* for both ROS and ΔΨM than has been measured among ETC mutant strains of *C. elegans* [8], [11]. This result is partly explained by the greater phenotypic variation observed *within C. elegans* mutant strains (e.g., see among-replicate variation in Figs. 2 and 3 in [8]) than within *C. briggsae* isolates; the source of this species difference is unclear. Our findings also mirror those of previous studies revealing extensive variation in life-history and metabolic traits among the same isolates [47], [52]. A good deal of this variation was related to the phylogeographic clade membership of particular isolates (Fig. 3; Table S1); this was especially true for ΔΨM (Fig. 3G; Table S2). *C. briggsae* is globally distributed [80] and both mitochondrial and nuclear genetic analyses consistently reveal the existence of distinct phylogeographic clades that are separated by latitude [52], [81]. These clades have experienced different population genetic histories [46], [52], [81] and show some evidence for local adaptation to temperature; e.g., Tropical clade isolates appear to have higher thermal maxima [53]. Consequently, divergence in mitochondrial phenotypes measured here may reflect clade-specific phylogenetic or selective histories. It is impossible to say without further study whether the among-clade differences in mitochondrial function measured here have an adaptive significance or are a non- or maladaptive consequence of genetic drift [52]; however, it is conceivable that Tropical *C. briggsae* isolates adaptively maintain low ΔΨM. Because *C. briggsae* is ectothermic, its biology will be driven strongly by environmental temperature. At higher temperatures, biological reactions such as those controlling electron transport and the subsequent production of ROS occur at higher rates [82]. The higher temperatures experienced by Tropical clade isolates may have thus subjected them to higher ROS levels during their evolutionary history. Reducing ROS production, especially from complex I, may be achieved by lowering ΔΨM by uncoupling oxidative phosphorylation [83], [84]. We may therefore hypothesize that Tropical *C. briggsae* have adaptively reduced their ΔΨM to counter increased ROS levels brought on by higher temperatures and perhaps exacerbated by *nad5Δ*-induced complex I inefficiency.

Although other traits appear to be influenced by both nuclear and mitochondrial genetic contributions, our data are consistent with the interpretation that much of the among-isolate variation in mitochondrial ROS and ΔΨM can be attributed to mitochondrial genome content rather than to nuclear divergence among isolates (Fig. 4; Table S4). In agreement with this finding, ROS level and ΔΨM were shown to be the best predictors among those tested of *nad5Δ* category in classification analyses (Table S2). Because mitochondrial electron transport relies upon the coordinated functioning of both mitochondrial and nuclear encoded ETC components, there is ample opportunity for epistatic interactions between mtDNA and nuclear genomes [67], [85]–[87]. A recent analysis of recombinant inbred lines generated from reciprocal crosses between *C. briggsae* isolates AF16 and HK104 provides indirect evidence for such mitochondrial-nuclear incompatibilities [45]. In agreement with this result, we observed extensive paternal transmission of mitochondria in other sets of *C. briggsae* mitonuclear hybrid strains (Materials and Methods). It could therefore have easily been the case that *C. briggsae* hybrids studied here bore no similarity to either parental isolate as a result of interpopulation hybrid breakdown. This is particularly true for our hybrid strains, both of which resulted from crosses between a Tropical and a Temperate isolate (Fig. 1). That the results of our hybrid strain analyses for ROS and ΔΨM are consistent with an additive effect of the mitochondrial genome suggests that the natural isolates used to generate the hybrids have either not experienced functional divergence for the relevant ETC gene products or have purged variants that generate deleterious epistatic interactions before they had the opportunity to create fixed polymorphisms between isolates [88]. An analysis of nucleotide diversity at non-synonymous versus synonymous codon positions (π_a_/π_s_) in ten mtDNA-encoded ETC genes among 22 *C. briggsae* natural isolates suggested that these genes are under purifying selection (π_a_/π_s_ <1 for all genes) and have not likely experienced functional divergence [52]. Patterns of diversity and divergence at nuclear-encoded ETC genes have not been analyzed in *C. briggsae* natural isolates. In either case, our results suggest that these particular hybrid strains will be valuable for future studies of mitochondrial function.

While results of the hybrid analyses showed that ROS and ΔΨM were influenced by mtDNA content, analyses of the natural *C. briggsae* isolates suggested that at least a portion of the among-isolate phenotypic variation may be associated with *nad5Δ* heteroplasmy level or other factors in linkage disequilibrium with *nad5Δ* (Fig. 3; Table S3). In particular, several traits exhibited non-linear relationships with isolate-specific *nad5Δ* level, a pattern in agreement with previous findings for these isolates [47]; however, the patterns are in most cases quite weak (Table S3; Fig. 3). Our results for highest-deletion isolate HK105 are of special note, however, since this isolate exhibits the highest ROS levels (Figs. 3 and 4) and the lowest reproductive fitness (Fig. 2A in Estes et al. 2011). It may be that HK105 (>50% deletion bearing genomes) has reached a threshold beyond which *nad5Δ* elicits deleterious effects; i.e., high ROS levels associated with extreme ETC dysfunction [26]. Congruent with this idea, HK105 also had the largest ratio of functional to non-functional organelles (Fig. 3H) and the smallest area of depolarized mitochondria (Fig. 3B) compared to other isolates. One interpretation of these data is that mitochondria with *nad5Δ* loads beyond ∼50% are unable to rescue functionality through mitochondrial fusion [89], [90] and the ensuing mitophagic degradation of highly impaired organelles increases the ratio of functional to non-functional organelles. A direct test of this hypothesis awaits development of techniques for simultaneously genotyping and phenotyping individual mitochondria.

It has been proposed that lowering mitochondrial ΔΨM may slow the rate of ROS production and help to alleviate oxidative stress – and perhaps extend lifespan [11], [83], [84]. Based on such studies and on the fact that ROS and ΔΨM were most important for classifying our *C. briggsae* natural isolates into *nad5Δ* categories (Table S2), we expected that a statistical relationship between these traits might emerge, but observed no such correlation (Figs. 3G, 3I). A caveat that prohibits further interpretation of this result is that ROS and ΔΨM levels were necessarily measured on different individual nematodes; estimates of each trait were therefore obtained from different sets of mitochondria (Materials and Methods). (Conversely, ΔΨM and morphology assays were conducted on the same individuals.) Unfortunately, to our knowledge, all ROS and ΔΨM probes utilize nearly identical fluorescent spectra, making simultaneous *in vivo* quantification of both traits impossible. It is noteworthy, however, that the results of our studies differ from those of Lemire et al. (2009), which showed that reduced ΔΨM was associated with increased lifespan across four classes of *C. elegans* longevity mutants. We find no obvious relationship between isolate-specific ΔΨM and lifespan measured in a previous study [47]. In particular, the long-lived PB800 isolate [47] was observed here to have a high ΔΨM (Fig. 2G). Further study would be required to understand why this isolate deviates from the strong pattern seen in *C. elegans* experimental strains. A possible explanation may lie in the nuclear genetic differences among the *C. briggsae* isolates used here; in contrast, Lemire (2009) studied *C. elegans* mutants on an otherwise common nuclear background.

Our analyses of mitochondrial phenotypes also identified traits that diagnose mitochondrial functionality. Apart from ROS, ΔΨM, and A_FP_ (combined area of the functional mitochondrial population), traits retained in classification trees all described some aspect of depolarized mitochondria (Table S2). In other words, descriptors of depolarized mitochondria provided more information about the *nad5Δ* frequency class to which natural isolates belonged than did descriptors of functional mitochondria. For example, although isolates did not differ in any aspect of functional mitochondrial shape (Table 1), traits describing non-functional mitochondrial shape (AR_N_, C_N_, and C_NV_) were retained in classification trees; these traits also showed especially obvious non-linear relationships to *nad5Δ* frequency (Fig. 3D–F). In particular, depolarized mitochondria tended to be larger (Fig. 3D) and more variable with respect to circularity (Fig. 3F) (and consequently less circular, Fig. 3E) in zero- and high-*nad5Δ* strains. Furthermore, although some traits describing the average number and combined area of the functional mitochondrial *population* were found to be (mostly non-linearly) related to *nad5Δ* frequency in our regression analyses (Table S3), traits describing *individual* functional mitochondria were never found to be associated with *nad5Δ* level in any analysis. Taken together, our results suggest that isolates with different *nad5Δ* levels are more variable with respect to their depolarized mitochondrial populations than to their polarized mitochondrial populations. Furthermore, we found that functionality (based on ΔΨM) is strongly associated with elongated organelles, whereas non-functionality is associated with circular (i.e., fragmented) organelles. This result is in agreement with previous findings [33], [38], [41], [90] and with the idea that damaged mitochondria lose ΔΨM and undergo fragmentation early in the cellular apoptosis process [32].

## Conclusions and Outlook

We have reported a novel analysis of subcellular processes in *C. briggsae* that, to our knowledge, provides the first explicit treatment of within-species natural variation in form and function of an organelle. Through the use of mitochondrial-nuclear hybrid lines, we demonstrated that mtDNA genotype is a strong driver of a portion of this natural variation. We also found evidence for complex associations between mitochondrial *nad5Δ* frequency and mitochondrial functioning (ROS and ΔΨM) and morphology traits. Although our study represents a major step forward in understanding natural variation in subcellular processes *in vivo*, additional work is required to move beyond correlative associations between mitochondrial genotypes and phenotypes to a direct determination of the genetic underpinnings of subcellular variation. Achieving this goal in our system would necessitate the simultaneous genotyping (e.g., through mitochondrial mRNA or DNA labeling) and phenotyping of individual mitochondria, which is not possible currently.

More generally, our study indicates that evolutionary approaches hold promise for advancing our knowledge of mitochondrial population dynamics and other cell-level processes. Although there is a long history of evolutionary analysis providing key insights into DNA-level genetic processes and organismal phenotypes, phenomena at more intermediate levels of biological organization (e.g., cellular, subcellular) have largely been overlooked by evolutionary biologists during the last century. Likewise, cell biology research rarely considers natural within-species variation. Evolutionary cell biology is an emerging and essentially untapped research area in need of both theoretical and empirical work. With regard to mitochondria, outstanding questions in this discipline include: what is the role of mitochondrial fission and fusion in purging deleterious heteroplasmic mtDNA mutations, how do epistatic interactions between nuclear and mtDNA subunits of ETC genes affect mitochondrial form and function and organismal fitness, and how do we analyze the mode and strength of selection on mitochondrial form and function? Together with the many other advantages to the *C. briggsae* system, its amenability to *in vivo* physiological studies and abundant among-isolate mtDNA variation suggest that this species will be a valuable natural model system for addressing such questions.

## Supporting Information

Table S1 **Effect of phylogenetic clade (df = 2) and strain nested within clade (df = 7) for mitochondrial form and function traits.** The F-ratio and degrees of freedom for nested analyses of variance for each trait are shown with *, **, and *** denoting p<0.05, 0.01, 0.001, respectively. Subscripts N and F indicate whether the measure refers to Non-functional or Functional mitochondria. Subscript P denotes that the measure refers to the entire mitochondrial population, rather than individual mitochondria. Subscript V denotes measures of average individual variance in that trait.
(XLSX)

Table S2 **Results of classification tree analysis. ROS and ΔΨM traits reflect average maximum fluorescence values.** Misclassification rate and R^2^ are the mean values from five separate runs of the classification tree using identical parameters (see text). (Top) The three phylogenetic clades of *C. briggsae* (Fig. 1) were used as grouping variables during tree construction. (Bottom) Four categories based on isolate-specific *nad5Δ* heteroplasmy level (Fig. 1) were used as grouping variables.
(XLSX)

Table S3 **Assigned labels of all mitochondrial traits measured for** ***C. briggsae*** **natural isolates.** The test statistic and adjusted r^2^ values for the best fit regression of each phenotype to *nad5Δ* frequency are given for each trait. Bold font identifies the nine traits retained in the classification tree analysis when using categories based on isolate-specific *nad5Δ* % (see Table S3). Italicized values indicate that a linear regression provided the best fit to *nad5Δ*%, while standard text denotes a quadratic relationship to *nad5Δ* %. *, **, and *** denote p<0.05, 0.01, 0.001, respectively. Subscripts N, F, and T indicate whether the measure refers to Non-functional, Functional, or Total mitochondria. Subscript P and V denote that the measure refers to the entire mitochondrial population (not individual mitochondria), or the average individual variance in that trait, respectively.
(XLSX)

Table S4 **Comparison of mitonuclear hybrid strains to parent strains.** The difference between the means of each pair of mitonuclear hybrids and their parental isolates are shown. Bolded traits exhibit significant differences among hybrid and parent strains. *, **, and *** denote p<0.05, 0.01, and 0.001 respectively (Tukey HSD, α = 0.05). AF = AF16, PB = PB800, HK = HK105.
(XLSX)
